# Differences in the prevalence of vitamin D deficiency and hip fractures in nursing home residents and independently living elderly

**DOI:** 10.1590/2359-3997000000109

**Published:** 2016-02-11

**Authors:** Alexander Shinkov, Anna-Maria Borissova, Lilia Dakovska, Jordan Vlahov, Lidia Kassabova, Dobrin Svinarov, Stefan Krivoshiev

**Affiliations:** 1 Department of Thyroid and Bone Mineral Diseases University Hospital of Endocrinology Medical University of Sofia Sofia Bulgaria Department of Thyroid and Bone Mineral Diseases, University Hospital of Endocrinology, Medical University of Sofia, Sofia, Bulgaria; 2 Central Laboratory of Therapeutic Drug Management and Clinical Pharmacology Alexander University Hospital Medical University of Sofia Sofia Bulgaria Central Laboratory of Therapeutic Drug Management and Clinical Pharmacology, Alexander University Hospital, Medical University of Sofia, Sofia, Bulgaria; 3 Department of Hemodialysis Queen Jovanna Hospital Medical University of Sofia Sofia Bulgaria Department of Hemodialysis, Queen Jovanna Hospital, Medical University of Sofia, Sofia, Bulgaria

**Keywords:** 25-hydroxyvitamin D, vitamin D deficiency, vitamin D insufficiency, elderly, nursing home residents

## Abstract

**Objective:**

: To compare the prevalence of vitamin D deficiency and fracture history in nursing home residents and community-dwelling elderly subjects and to explore the association of vitamin D levels with various characteristics.

**Materials and methods:**

Sixty-six nursing home residents and 139 community-dwelling elderly subjects participated. Marital status, medical history, medication including vitamin D supplements, smoking, past fractures were assessed. Weight and height were measured and body mass index calculated. Serum 25-hydroxyvitamin D (25-OHD), PTH, Ca, phosphate, creatinine and eGFR were determined.

**Results:**

: In the nursing home residents 25-OHD was lower (17.8 nmol/l, [9.4-28.6] *vs*. 36.7 nmol/l, [26.9-50], p < 0.001), PTH was higher (5.6 pmol/l, [3.9-8.9] *vs*. 4.7 pmol/l [3.6-5.8], P = 0.003) and 25-OHD deficiency was more prevalent (65.2% [53.7-76.7] *vs*. 22.3% [15.4-29.2], p < 0.001) as was elevated PTH (23% [12.8-33] *vs*. 5.8% [2-10], p = 0.001). 25-OHD correlated negatively with PTH (institutionalized r = -0.28, p = 0.025 and community-dwelling r = -0.36, p < 0.001). Hip fractures were reported by 8% of the residents and 2% of the independent elderly. The only predictor for hip fracture was elevated PTH (OR = 7.6 (1.5-36.9), p = 0.013).

**Conclusion:**

: The prevalence of vitamin D deficiency and secondary hyperparathyroidism was high in the institutionalized subjects. Hip fracture risk was associated with elevated PTH and not directly with vitamin D levels or the residency status.

## INTRODUCTION

The term Vitamin D designates a group of steroid compounds that are essential for human health (1). The major source of vitamin D for the humans is the sun exposure of the skin (2). Provitamin D is first synthesized from 7-dehydrocholesterol and then converted to vitamin D3 (cholecalciferol). Choleclaciferol undergoes a two-step conversion first into 25-hydroxyvitamin D (25-OHD) and then into 1,25-Dihydroxyvitamin D [1,25(OH)2D] with 25-OHD serum levels most closely reflecting the body vitamin D state. Vitamin D synthesis depends on a number of factors like geographic latitude, climate, clothing, individual sun exposure, air pollution, sunscreen use, gender, obesity (3,4). There are data that skin capacity for cholecalciferol synthesis diminishes with age (5,6). The insufficient skin vitamin D synthesis or food intake lead to vitamin D deficiency, which is common in large parts of the World on both sides of the Equator (7). Serious health-related implications have been attributed to low vitamin D levels – osteomalacia and increased fracture risk, muscle dysfunction in the elderly subjects, increased cardiovascular morbidity and higher rate of some cancers and autoimmune diseases (8-10). Though the problem affects all ages, nursing home elderly residents are among the groups with the highest risk of vitamin deficiency or its consequences (11-13). Factors that determine the latter are malnutrition, decreased mobility and sun exposure, as well as the physiologic changes in vitamin D synthesis with ageing. Low vitamin D levels have been associated with increased hip fractures with institutionalized subjects being the most affected (14). Moreover, the authors of a recent analysis reached the conclusion that the institutionalized elderly are the group most likely to benefit from systemic vitamin D supplementation (15).

The aim of the study was to compare the prevalence of vitamin D deficiency and self-reported fracture history among nursing home residents and community-dwelling elderly subjects and explore the association of vitamin D levels with the gender, body mass index and the prevalence of secondary hyperparathyroidism (SHPT).

## MATERIALS AND METHODS

The study was a part of a cross-sectional screening study of the prevalence of vitamin D deficiency and the most common endocrine disorders in the Bulgarian population, carried out in January and February 2012 in 12 towns and the adjacent rural areas. Details on the initial study cohort recruitment and characteristics and the general vitamin D state of the population are published elsewhere (16).

Sixty-six nursing home residents from four separate nursing homes participated in the study. The control group consisted of the age matched community-dwelling elderly subjects from the same towns, n = 139. None of the study subjects was bed-ridden or reported recent long-term immobilization. All subject signed an informed consent form, approved by the local Ethics committee at the University Hospital of Endocrinology in Sofia. The participants answered a structured interview with questions concerning marital status, past or present disorders, current medication including vitamin D-containing preparations, smoking, fracture history. Body weight and height were measured and body mass index (BMI) was calculated by the standard formula. Fasting venous blood was sampled and serum was stored at -20 C for serum 25-OHD, PTH, Ca, inorganic phosphate (iP), creatinine, and TSH determination. eGFR was calculated by the CKD-EPI formula: GFR = 175 × standardized Scr−1.154 × age−0.203 × 1.212 [if black] × 0.742 [if female], where GFR is expressed as mL/min/1.73 m^2^ of body surface area 41 and Scr is expressed in mg/d (17).

The fractures were reported as any fractures and separately for the hip, Colles’ and vertebral fractures.

### Laboratory evaluation

25-OHD was determined by liquid chromatography-tandem mass spectrometry (LC-MS/MS) utilizing extraction with hexane. d325D_3_ was applied as an internal standard with reaction monitoring for the respective m/z transitions: 401→383 for 25D_3_, 413→395 for 25D_2_, and 404→386 for d325D_3_. The method complies with the FDA required selectivity, matrix effect, accuracy, precision within 7.5% and extraction recoveries averaging 57-73%. Linearity was within 3.0-300.0 nmol/L, R^2 ^> 0.99 (18). Based on the measured 25-OHD levels, three categories of subjects were defined: sufficient – > 50 nmol/l, deficient – 25-50 nmol/l and severely deficient – < 25 nmol/l and the proportion of subjects with severe deficiency and sufficiency were explored.

PTH was determined by chemiluminesce with an Immunoradiometric assay (IRMA) Access2/DxI, reference range 1.3-9.3 pmol/l. TSH was measured as a possible confounder and was determined by a two-site immunoenzymatic “sandwich” assay (UniCel^®^ DxC 660i System, Beckman Coulter, Access HYPER sensitive hTSH) with a reference range between 0.34-5.60 mIU/l. Serum calcium, inorganic phosphate, creatinine and alkaline phosphatase were measured by an automated analyzer.

### Statistical analysis

Statistical analysis was performed by SPSS for Windows v.13 (SPSS Corp, Chicago, Il). All numerical data were presented as means and standard deviations or median and interquartile range. Normality of distribution was explored by Kolmogorov-Smirnov test. Student t-test was used to compare the difference between different categories of normally distributed continuous variables. Mann-Whitney U test and median test were applied for non-normally distributed variables. Proportions were expressed as percent and 95% confidence intervals and compared by Chi-square and Fisher’s exact test. A logistic regression was performed to explore the effect of multiple factors on the prevalence of vitamin D deficiency. Spearman’s rho was used to measure correlations between the studied continuous variables. All correlations of 25(OH)D were performed after controlling for age and gender. All tests were two-sided, with a level of significance at p < 0.05.

## RESULTS

Eight of the community-dwelling subjects and none of the nursing home residents reported vitamin D supplement use. The reported average daily intake was between less than 100 and 400 IU. The 25-OHD levels of the supplement-users did not differ from the group mean value and therefore were not excluded from the analysis. Of the community-dwelling subjects, 69.8% [62.2-77.4] lived with a spouse *versus* 9.1% [2.2-16] of the nursing home residents.

The nursing home residents were significantly older than the community-dwelling subjects and all further analyses were adjusted for age. The two groups differed in the female-to-male ratio (Table 1) and were first stratified by gender. As the main studied characteristics did not demonstrate significant inter-gender differences (data not shown), all further analyses were done without gender differentiation of the subjects. The correlations and the regression models were adjusted for gender.

**Table 1:** Comparison of the studied characteristics of the community-dwelling elderly and the nursing home residents

Characteristic	Nursing home resident (n = 66)	Community-dwelling (n = 139)	p
Age (years)	74.5 [69.8-78]	64 [61-70]	< 0.001
Female/Male	26 (39%)/40 (61%)	91 (65%)/48 (35%)	0.001
BMI (kg/m^2^)	26.0 ± 4.5	28.9 ± 4.9	< 0.001
25(OH)D (nmol/l)	17.8 [9.4-28.6]	36.7 [26.9-50]	< 0.001
TSH (mIU/l)	1.68 [1.16-2.94]	2.16 [1.28-3.55]	0.27
PTH (pmol/l)	5.6 [3.9-8.9]	4.7 [3.6-5.8]	0.003
Ca (mmol/l)	2.32 ± 0.18	2.41 ± 0.12	0.001
iP (mmol/l)	1.12 ± 0.18	1.22 ± 0.18	0.001
AlP (IU/l)	77.5 [65-103.3]	65 [52-78]	< 0.001
eGFR (mL/min/1.73 m^2^)	73.7 ± 21.1	76.8 ± 15.3	0.27
Married (%)	9.1% [2.2-16]	69.8% [62.2-77.4]	< 0.001

The comparison of the major group characteristics between the community-dwelling elderly and the nursing home residents is presented in Tables 1 and 2.

We calculated eGFR in order to rule out a possible effect of chronic kidney disease. eGFR of the subjects did not differ between the two groups and correlated negatively with the age (Community-dwelling elderly r = -0.39, p < 0.001; residents r = -0.46, p < 0.001), but not with the PTH levels (Community-dwelling elderly r = -0.03, p = 0.70; residents r = -0.06, p = 0.66). It correlated negatively with 25-OHD in the institutionalized subjects (Table 3). The odds ratio for a vitamin D deficiency among the nursing home residents as compared to the community-dwelling elderly subjects was 6.3 (95%CI 3.1-12.6) in a model adjusted for age, gender, eGFR and TSH.

**Table 3:** Correlations of 25-OHD levels with the other studied parameters in the two groups, after controlling for age and gender

Parameter	Nursing home residents (n = 66) Spearman Rho, p	Community-dwelling elderly (n = 139) Spearman Rho, p
PTH	-0.28, p = 0.025	-0.36, p < 0.001
sCa	0.06, p = 0.64	-0.04, p = 0.62
iP	-0.01, p = 0.94	-0.04, p = 0.63
AlP	-0.24, p = 0.06	-0.02, p = 0.84
eGFR	-0.25, p = 0.048	0.09, p = 0.31

### Fracture prevalence

The prevalence of any fractures or Colles’ fractures did not differ between the two groups (Table 2). Only four subjects reported known history of vertebral fractures. The cases were distributed equally between the nursing home residents and the independent elderly. No further analysis was done due to the low count. The prevalence of hip fractures was higher in the nursing home residents, but the small count does not permit estimation of the statistical significance of the difference. The mean 25-OHD level was lower in the subjects with hip fractures (21.1 ± 5.4 *vs*. 33.4 ± 1.3, p = 0.064), but not in those with any fracture or with Colles’ fractures.

**Table 2:** Proportions of vitamin D sufficiency and severe deficiency, fracture prevalence and secondary hyperparathyroidism in the community-dwelling elderly and the nursing home residents

	Nursing home residents (n = 66) % [95%CI]	Community-dwelling elderly (n = 139) % [95%CI]	p
Deficiency	65.2% [53.7-76.7]	22.3% [15.4-29.2]	< 0.001
Sufficiency	4.5% [0-9.5]	25.4% [17.4-31.6]	< 0.001
All fractures	18.2% [8.9-27.5]	17.3% [11-23.6]	NS
Hip fractures	7.6% [1-14]	2.2% [0-4.6]	-
Colles’ fractures	6,1% [0-12]	5.8% [2-9.7]	NS
Elevated PTH	23% [12.8-33]	5.8% [2-10]	0.001

### Secondary hyperparathyroidism

In both groups the 25-OHD levels correlated negatively with PTH, but with none of the other variables (Table 3). SHPT, expressed as an elevated PTH, was found in 23 (13.7%) of the subjects. It was more prevalent in the home residents than in the community-dwelling subjects (Table 2). PTH was elevated in 22% of the subjects with vitamin D deficiency, in 5% of those with insufficiency and in none of the ones with sufficiency, p < 0.001. The hip fractures were more prevalent in the subjects with elevated PTH (13% *vs*. 3%, p = 0.048) but the count was too small for definite conclusions. There was no difference in the all fracture and Colles’ fracture prevalence among the subjects with normal or elevated PTH. In a logistic regression model adjusted for age, gender, 25-OHD level, eGFR, TSH, resident/not-resident the only factor significantly affecting the risk of a hip fracture was the elevated PTH with an OR = 7.6 (1.5-36.9), p = 0.013.

## DISCUSSION

In our winter study we compared community-dwelling elderly and institutionalized subjects and observed significantly lower vitamin D levels and higher prevalence of vitamin D deficiency in the latter – 95% *vs*. 75%. Severe vitamin D deficiency was found in every third resident. Our results do not differ from those reported by other authors from various geographic regions. Maeda and cols. for instance measured similar winter levels and difference between community-dwelling elderly and nursing home residents (19). Their study was carried in Brazil, at latitude of 23° S. The locations included in our study lie between 42°40’ and 43°50’ N and much lower UVB should be expected. Pilz and cols. also studied a large group of nursing home residents in winter in Austria and found comparable median 25-hydroxyvitamin levels of 17.5 nmol/l. Furthermore, 93% of the nursing home residents studied by them had 25-OHD below 50 nmol/l, which is very similar to the proportion we observed (13).

Various factors contribute to the low vitamin D state in the institutionalized elderly. Similar to our data, a Romanian team reported recently that none of the nursing home residents whom they studied received vitamin D supplementation (20). The vitamin D supplementation among the community-dwelling elderly in our study was also very low and cannot explain the difference by itself. Vitamin D food fortification is not mandatory in Bulgaria and there is virtually no fortified foodstuff on the market. Fish consumption is also low and we can assume that for the majority of the population the alimentary vitamin D intake is generally scarce.

The sun exposure therefore might be the major factor that differentiates the two studied groups. There are few data in the literature researching the topic of sun exposure and vitamin D in nursing home residents. Nashimoto and cols. reported a distinct association between 25-hydroxyvitamin D levels and hours of sun exposure in institutionalized elderly (21). A pivotal study performed by Webb and cols. demonstrated a marked difference in the sun exposure between independently living elderly and nursing home residents (22). Moreover, the difference persisted irrespective of the mobility level of the residents. The lower sun exposure in residents has been linked also to a flatter all-year vitamin D curve (19). The sun exposure of our subjects was limited by the typically cold winter weather. The time the residents spent outdoors was further restricted by the anxiety associated to falls and possible fractures, maintaining apparently a vicious circle: anxiety – less outdoor time – less sunlight exposure and vitamin D – more fractures – more anxiety. The issue has been described typically among people recovering from a hip fracture (23). The fear of falls affects their self-confidence, restricts their mobility and leads to activity avoidance. An inverse association with low vitamin D levels as a consequence and hip fractures as a factor might therefore exist in certain subjects.

The hip fracture rate in the institutionalized elderly and in those with severe vitamin D deficiency was marginally higher than among the independent elderly or those with higher 25-hydroxyvitamin D levels. Similarly to the results of other authors, 25-hydroxyvitamin D was lower in the subjects reporting hip fractures (14). On the other hand, the unadjusted odds ratio for a hip fracture was not significantly increased for any of the two factors (low vitamin D or nursing home residency) as predictors. Instead, the only significant predictor for a hip fracture was SHPT. The latter was related both to resident status and vitamin D levels. As observed previously (18,21,24), SHPT is by itself an independent risk factor for hospitalization and/or death in the elderly. The prevalence of SHPT expressed as an elevated PTH and higher alkaline phosphatase was higher among the nursing home residents in our cohort. The latter presented also with lower serum calcium and inorganic phosphate, presumably resulting from the vitamin D deficiency. It has been debated whether vitamin D deficiency is an independent factor for fragility fractures in the elderly. Low vitamin D-induced reduction in muscle strength and tone contribute to susceptibility to falls and consequently – fractures (25). It was recently demonstrated also that 25-hydroxyvitamin D levels were inversely correlated with time to first fall in the elderly (26). As fall frequency is important for hip fracture occurrence, it has been shown that institutionalized subjects who have suffered hip fracture are more prone to falling than independently living hip fracture patients (27). Lower BMD has been reported in subjects with vitamin D deficiency as well (12,28). Nevertheless it is possible that the association of vitamin D levels and bone fragility does not result directly from low vitamin D, but is mediated by the SHPT, as demonstrated by Arabi and cols. (29). The reduced serum calcium and phosphate might further compromise bone mineralization and strength.

We observed no association of vitamin D or PTH levels or fracture prevalence with the BMI. No association was found also with any other reported fracture type. However the self-reported vertebral fracture prevalence was much lower than expected and underreporting might have occurred (28). Three subjects reported both Colles’ and hip fractures, all of them being nursing home residents with severe vitamin D deficiency. PTH was elevated in two of them. The low count requires cautious interpretation, but published data point to an increased risk of subsequent upper extremity fracture in hip fracture patients with vitamin D deficiency (30). Moreover, the reported association was independent of bone mineral density.

Advanced age is associated with a declining renal function, another causal factor for SHPT in the elderly (31,32). Its role for the bone metabolism in the ageing man is not yet well defined (32). Renal function as measured by eGFR in our study correlated inversely with age but not with PTH or 25-OHD. Furthermore, eGFR did not predict hip fracture, vitamin D deficiency or elevated PTH. Therefore we assumed that impaired renal function might not affect our conclusions.

Our study has several limitations. In the first place, the number of subjects with fractures was relatively low, thus limiting the statistical power of the analysis. Second, though it is well accepted that the UV radiation in Bulgaria in late autumn and winter is low, the study could benefit from an assessment of the sun exposure of the studied subjects. Third, the fractures were self-reported, which precludes the assessment of vertebral fractures and also introduces imprecision concerning the time since the fracture. The temporal difference between the time of the fractures and the study limits the conclusiveness of the results. We had no data on falls that may be an important mediator between vitamin D and fractures. The cross-sectional design on the other hand does not permit causality inferences.

In conclusion, we observed a high prevalence of vitamin D deficiency and SHPT in the studied nursing home residents. Hip fracture risk was associated with the elevated PTH and not directly to the vitamin D levels. Since vitamin D deficiency is the major cause of SHPT in the elderly, sufficient vitamin D supplementation should be recommended for the nursing home residents for whom the evidence for hip fracture reduction with supplementation is compelling (15).

## References

[1] . Hossein-Nezhad A, Holick MF. Vitamin D for health: a global perspective. Mayo Clin Proc. 2013;88:720-55. 10.1016/j.mayocp.2013.05.011 PMC3761874 23790560

[2] . Holick MF. Vitamin D, sunlight and cancer connection. Anticancer Agents Med Chem. 2013;13:70-82. 23094923

[3] . Freedman DM, Cahoon EK, Rajaraman P, Major JM, Doody MM, Alexander BH, et al. Sunlight and other determinants of circulating 25-hydroxyvitamin D levels in black and white participants in a nationwide U.S. study. Am J Epidemiol. 2013;177:180-92. 10.1093/aje/kws223 PMC3590034 23292956

[4] . Salamone LM, Dallal GE, Zantos D, Makrauer F, Dawson-Hughes B. Contributions of vitamin D intake and seasonal sunlight exposure to plasma 25-hydroxyvitamin D concentration in elderly women. Am J Clin Nutr. 1994;59:80-6. 10.1093/ajcn/59.1.80 8279408

[5] . Godar DE, Pope SJ, Grant WB, Holick MF. Solar UV doses of young Americans and vitamin D3 production. Environ Health Perspect. 2012;120:139-43. 10.1289/ehp.1003195 PMC3261929 21852226

[6] . MacLaughlin J, Holick MF. Aging decreases the capacity of human skin to produce vitamin D3. J Clin Invest. 1985;76:1536-8. 10.1172/JCI112134 PMC424123 2997282

[7] . van Schoor NM, Lips P. Worldwide vitamin D status. Best Pract Res Clin Endocrinol Metab. 2011;25:671-80. 10.1016/j.beem.2011.06.007 21872807

[8] . Park S, Lee BK. Vitamin D deficiency is an independent risk factor for cardiovascular disease in Koreans aged ≥ 50 years: results from the Korean National Health and Nutrition Examination Survey. Nutr Res Pract. 2012;6:162-8. 10.4162/nrp.2012.6.2.162 PMC3349039 22586506

[9] . Semba RD, Houston DK, Bandinelli S, Sun K, Cherubini A, Cappola AR, et al. Relationship of 25-hydroxyvitamin D with all-cause and cardiovascular disease mortality in older community-dwelling adults. Eur J Clin Nutr. 2010;64:203-9. 10.1038/ejcn.2009.140 PMC3277831 19953106

[10] . Schierbeck LL, Rejnmark L, Tofteng CL, Stilgren L, Eiken P, Mosekilde L, et al. Vitamin D deficiency in postmenopausal, healthy women predicts increased cardiovascular events: a 16-year follow-up study. Eur J Endocrinol. 2012;167:553-60. 10.1530/EJE-12-0283 22875588

[11] . Kuwabara A, Himeno M, Tsugawa N, Kamao M, Fujii M, Kawai N, et al. Hypovitaminosis D and K are highly prevalent and independent of overall malnutrition in the institutionalized elderly. Asia Pac J Clin Nutr. 2010;19:49-56. 20199987

[12] . Kruavit A, Chailurkit LO, Thakkinstian A, Sriphrapradang C, Rajatanavin R. Prevalence of vitamin D insufficiency and low bone mineral density in elderly Thai nursing home residents. BMC Geriatr. 2012;12:49. 10.1186/1471-2318-12-49 PMC3490934 22938528

[13] . Pilz S, Dobnig H, Tomaschitz A, Kienreich K, Meinitzer A, Friedl C, et al. Low 25-hydroxyvitamin D is associated with increased mortality in female nursing home residents. J Clin Endocrinol Metab. 2012;97:E653-7. 10.1210/jc.2011-3043 22319037

[14] . Holvik K, Ahmed LA, Forsmo S, Gjesdal CG, Grimnes G, Samuelsen SO, et al. Low serum levels of 25-hydroxyvitamin D predict hip fracture in the elderly: a NOREPOS study. J Clin Endocrinol Metab. 2013;98:3341-50. 10.1210/jc.2013-1468 23678033

[15] . Bolland MJ, Grey A, Gamble GD, Reid, IR. The effect of vitamin D supplementation on skeletal, vascular, or cancer outcomes: a trial sequential meta-analysis. Lancet Diabetes Endocrinol. Published Online: 24 January 2014. 10.1016/S2213-8587(13)70212-2 24703049

[16] . Borissova AM, Shinkov A, Vlahov J, Dakovska L, Todorov T, Svinarov D, et al. Vitamin D status in Bulgaria--winter data. Arch Osteoporos. 2013;8:133. 10.1007/s11657-013-0133-4 23526032

[17] . Levey AS, Stevens LA, Schmid CH, Zhang YL, Castro AF 3rd, Feldman HI, et al. A new equation to estimate glomerular filtration rate. Ann Intern Med. 2009;150:604-12. 10.7326/0003-4819-150-9-200905050-00006 PMC2763564 19414839

[18] . Shabir GA. Validation of high-performance liquid chromatography methods for pharmaceutical analysis. Understanding the differences and similarities between validation requirements of the US Food and Drug Administration, the US Pharmacopeia and the International Conference on Harmonization. J Chromatogr A. 2003;987:57-66. 10.1016/s0021-9673(02)01536-4 12613797

[19] . Maeda SS, Saraiva GL, Hayashi LF, Cendoroglo MS, Ramos LR, Correa Mde P, et al. Seasonal variation in the serum 25-hydroxyvitamin D levels of young and elderly active and inactive adults in Sao Paulo, Brazil: The Sao PAulo Vitamin D Evaluation Study (SPADES). Dermatoendocrinol. 2013;5:211-7. 10.4161/derm.24476 PMC3897593 24494057

[20] . Mocanu V,Vieth R. Three-year follow-up of serum 25-hydroxyvitamin D, parathyroid hormone, and bone mineral density in nursing home residents who had received 12 months of daily bread fortification with 125 mug of vitamin D3. Nutr J. 2013;12:137. 10.1186/1475-2891-12-137 PMC3874673 24120120

[21] . Nashimoto M, Nakamura K, Matsuyama S, Hatakeyama M, Yamamoto M. Hypovitaminosis D and hyperparathyroidism in physically inactive elderly Japanese living in nursing homes: relationship with age, sunlight exposure and activities of daily living. Aging Clin Exp Res. 2002;14:5-12. 10.1007/BF03324411 12027153

[22] . Webb AR, Pilbeam C, Hanafin N, Holick MF. An evaluation of the relative contributions of exposure to sunlight and of diet to the circulating concentrations of 25-hydroxyvitamin D in an elderly nursing home population in Boston. Am J Clin Nutr. 1990;51:1075-81. 10.1093/ajcn/51.6.1075 2349922

[23] . Jellesmark A, Herling SF, Egerod I, Beyer N. Fear of falling and changed functional ability following hip fracture among community-dwelling elderly people: an explanatory sequential mixed method study. Disabil Rehabil. 2012;34:2124-31. 10.3109/09638288.2012.673685 22536796

[24] . Premaor MO, Scalco R, da Silva MJ, Furlanetto TW. Secondary hyperparathyroidism is associated with increased risk of hospitalization or death in elderly adults living in a geriatric institution. Gerontology. 2009;55:405-10. 10.1159/000227760 19571528

[25] . Ringe JD. The effect of Vitamin D on falls and fractures. Scand J Clin Lab Invest Suppl. 2012;243:73-8. 10.3109/00365513.2012.681965 22536766

[26] . Rothenbacher D, Klenk J, Denkinger MD, Herbolsheimer F, Nikolaus T, Peter R, et al. Prospective evaluation of renal function, serum vitamin D level, and risk of fall and fracture in community-dwelling elderly subjects. Osteoporos Int. 2014;25:923-32. 10.1007/s00198-013-2565-5 24221451

[27] . Formiga F, Lopez-Soto A, Duaso E, Ruiz D, Chivite D, Perez-Castejon JM, et al. Differences in the characteristics of elderly patients suffering from hip fracture due to falls according to place of residence. J Am Med Dir Assoc. 2007;8:533-7. 10.1016/j.jamda.2007.06.007 17931578

[28] . Kuchuk NO, Pluijm SM, van Schoor NM, Looman CW, Smit JH, Lips P. Relationships of serum 25-hydroxyvitamin D to bone mineral density and serum parathyroid hormone and markers of bone turnover in older persons. J Clin Endocrinol Metab. 2009;94:1244-50. 10.1210/jc.2008-1832 19158198

[29] . Arabi A, Baddoura R, Awada H, Salamoun M, Ayoub G, El-Hajj Fuleihan G. Hypovitaminosis D osteopathy: is it mediated through PTH, lean mass, or is it a direct effect? Bone. 2006;39:268-75. 10.1016/j.bone.2006.01.140 16495164

[30] . Di Monaco M, Castiglioni C, Vallero F, Di Monaco R, Tappero R. Vitamin D depletion in hip fracture women is associated with the occurrence of simultaneous upper limb fractures independently of bone mineral density. Eur Geriatr Med. 2014;5:14-7.

[31] . Starr JM, Deary IJ. Renal function in a narrow-age cohort of adults at 79 and 87 years. Age Ageing. 2010;39:750-2. 10.1093/ageing/afq103 20823126

[32] . Van Pottelbergh G, Mathei C, Vaes B, Adriaensen W, Gruson D, Degryse JM. The influence of renal function on vitamin D metabolism in the very elderly. J Nutr Health Aging. 2013;17:107-11.10.1007/s12603-012-0094-023364486

